# Vitamin D and vitamin D receptor polymorphism in Asian adolescents with primary dysmenorrhea

**DOI:** 10.1186/s12905-023-02569-9

**Published:** 2023-08-05

**Authors:** Ainur Donayeva, Ainur Amanzholkyzy, Roza Nurgaliyeva, Gulnara Gubasheva, Samat Saparbayev, Dinmukhamed Ayaganov, Aiman Kaldybayeva, Ibrahim A. Abdelazim, Mohamed M. Farghali

**Affiliations:** 1 Department of Normal Physiology, West Kazakhstan Marat Ospanov Medical University, Aktobe, Kazakhstan; 2 Department of Obstetrics and Gynecology №2, West Kazakhstan Marat Ospanov Medical University, Aktobe, Kazakhstan; 3 Department of Neurology, West Kazakhstan Marat Ospanov Medical University, Aktobe, Kazakhstan; 4 Department of Obstetrics and Gynecology, Faculty of Medicine, Ain Shams University, Cairo, Egypt

**Keywords:** Vitamin D, Vitamin D receptor, Asian, Adolescents, Primary dysmenorrhea

## Abstract

**Background:**

The expression of vitamin D receptor in the normal endometrium and ovaries supports the role of vitamin D in local immunity and inflammatory cytokines regulation.

**Objective:**

This study aimed to detect the relation between serum 25(OH)D and primary dysmenorrhea in Asian Adolescents.

**Methods:**

Two hundred and five (205) adolescents complaining of primary dysmenorrhea (study group) were compared in this prospective study to matched controls (210 controls) after informed consent following the Helsinki Declaration.

After thorough evaluation, including a thorough history and pelvic ultrasound examination, blood samples were collected from the studied adolescents to measure serum 25(OH)D and for vitamin D receptor TaqI (rs731236) genotyping.

The studied adolescents’ data were analyzed using the Pearson’s correlation to detect the relation between serum 25(OH)D and primary dysmenorrhea (primary outcome). The secondary outcome measures the odds of primary dysmenorrhea in Asian adolescents with vitamin D receptor TaqI (rs731236) polymorphism.

**Results:**

The serum 25(OH)D was significantly lower in the studied-dysmenorrhea group compared to controls (16.17 ± 7.36 versus 17.65 ± 6.36 ng/ml, respectively), (*P* = 0.01). The correlation analysis showed a significant negative correlation between the serum 25(OH)D and visual analogue scale of dysmenorrhea (r = -0.9003, *P* < 0.0001).

The studied-dysmenorrhea cases with vitamin D receptor T/t and t/t genotypes had significantly lower serum 25(OH)D (16.7 ± 8.05 and 14.4 ± 4.1 ng/ml, respectively) compared to controls (18.97 ± 6.7 and 21.4 ± 2.45 ng/ml, respectively), (*P* = 0.02 and 0.004, respectively).

The VDR T/t and t/t polymorphisms significantly increase the odds of primary dysmenorrhea (OR 1367.2, *P* < 0.0001 and OR 106.2, *P* = 0.001, respectively).

**Conclusion:**

The serum 25(OH)D was significantly lower in the studied-dysmenorrhea group compared to controls. The studied-dysmenorrhea cases with VDR T/t and t/t TaqI genotypes had significantly lower serum 25(OH)D compared to controls. The VDR T/t and t/t polymorphisms significantly increase the odds of primary dysmenorrhea.

## Background

Primary dysmenorrhea (PD) is painful menstruation without pelvic pathology [1]. The PD starts before the menses and lasts 8–72 h [2].

The PD affects 16–91% of reproductive-age women [3]. A systematic review reported a 71% prevalence of PD in Iran [4]. Dysmenorrhea negatively affects daily activities and work productivity [5].

Increased uterine prostaglandins (PGDs) is the most accepted aetiology of PD [6, 7]. The PGDs increase the amplitude and tone of uterine contractions [7]. Therefore, the non-steroidal anti-inflammatory drugs (NSAIDs) which inhibit the PGDs synthesis are the first-line treatment of PD [8]. The NSAIDs are usually associated with increased risk of gastric ulcers and gastrointestinal bleeding [9, 10]. Therefore, the relief of PD with other therapeutic options could be helpful and limit the use of NSAIDs.

Low serum vitamin D (Vit. D) was reported during the luteal phase of the menstrual cycle [11], and a systematic review reported Vit. D deficiency in severe PD [12].

*Agic *et al., reported expression of VDR in the normal endometrium and ovaries [13], which supports the role of Vit. D in local immunity and inflammatory cytokines regulation [14].

Vit. D metabolites [i.e., 25(OH)D and 1,25(OH)_2_D] reduce the levels of inflammatory cytokines and uterine PGDs [15, 16], and the Vit. D supplementation could reduce the severity of PD [17, 18].

The Vit. D receptor (VDR) gene is a protein-coding gene, located on 12q13.11 and expresses a receptor rs731236 that influences the Vit. D binding ability. Any mutation in the VDR gene may result in defective Vit. D binding and subsequent Vit. D deficiency [14].

Considering the anti-inflammatory role of Vit. D and the previous promising results of Vit. D in dysmenorrhea [17, 18], it’s expected that 25(OH)D deficiency and/or VDR TaqI (rs731236) polymorphism could increase the severity of PD.

Therefore, this study was designed based on a hypothesis that 25(OH)D deficiency and/or VDR TaqI (rs731236) polymorphism in Asian adolescents could increase the severity of PD.

## Methods

Two hundred and five (205) adolescents between 12–18 years-old complaining of PD [study group (dysmenorrhea group)] were compared in this prospective study to matched controls (210 adolescents without dysmenorrhea) of the same age and ethnic characteristics.

Adolescents (study and controls = 415) were recruited for this comparative prospective study, which was conducted from January 2021 to November 2022 (from 43 schools of Aktobe-Kazakhstan) after approval No. 10 dated 04 October 2020 from the ethics committee of West Kazakhstan Medical University (WKMU).

Adolescents were included in this study after informed consent from the adolescents themselves and their parents or legal guardians following the Helsinki Declaration.

The studied adolescents were evaluated thoroughly and examined by an adolescent gynecologist according to the hospital’s protocol.

After a thorough history, the studied adolescents’ weight and height were measured to calculate the body mass index (BMI).

A trans-abdominal pelvic ultrasound was done for the studied adolescents by an expert sonographer blinded to the adolescents’ data using the trans-abdominal convex probe (Samsung HS40, Samsung Co., Korea) to exclude any pelvic pathology.

Inclusion criteria: the study group (205 adolescents) includes adolescents between 12–18 years old, complaining of PD [visual analogue scale (VAS) ≥ 5] for ≥ one year since their menarche, with BMI < 30 kg/m^2^ and regular menstrual cycle (every 21–35 days). Control group (210 adolescents) includes adolescents between 12–18 years old, without dysmenorrhea for ≥ one year since their menarche, with BMI < 30 kg/m^2^ and regular menstrual cycle.

Exclusion criteria include adolescents < 12 or > 18 years old, with BMI > 30 kg/m^2^, pelvic organs anomalies (i.e., including anomalies of the genital and/or urinary tracts) or pelvic pathology (i.e., fibroid uterus and/or ovarian cyst or mass), previous pelvic surgery, neurological or psychiatric disorders or received exogenous hormonal therapy within the last year [19].

The BMI was calculated from the studied adolescents’ body weight and their height (kg/m^2^) [20, 21].

The WHO considered 18.5–24.9 kg/m^2^ normal BMI, 25–29.9 kg/m^2^ overweight, and > 30 kg/m^2^ obesity class-I [22].

The visual analogue scale (VAS) was used to assess the severity of the studied adolescents’ dysmenorrhea (0 is the lowest VAS and means no pain, while 10 is the highest VAS and means unbearable pain [23].

Blood samples were collected from the studied adolescents to measure the serum 25(OH)D and for VDR TaqI (rs731236) genotyping.

The serum 25(OH)D level was measured using an enzyme-linked immunosorbent assay (Ottignies-Louvain-la-Neuve., Belgium) [14].

Serum 25(OH)D between 20–40 ng/mL was considered normal, while serum 25(OH)D < 20 ng/mL was considered 25(OH)D deficiency according to the endocrine society clinical guideline [24].

The blood samples collected from the studied adolescents were used for DNA extraction using the standard salting out method [14], followed by VDR TaqI genotyping.

The TaqI genotyping was performed using polymerase chain reaction (PCR) and amplification of the targeted region.

Both TaqI wild-type and variant were amplified to produce the same product size (i.e., 148 bp) of the allele-specific primer design [14].

The sequences of the primers covering the targeted gene region were 50-CAGGACGCC GCGCTGATT-30 (forward primer for wild type), 50-CAGGACGCCGCGCTGATC-30 (forward primer sequence for variant allele) and 50-CCTCATTGAGGCTGCGCAG-30 (reverse common primer) [14].

The PCR was optimized after 30 cycles of denaturation at 94°C (5 min.), second denaturation at 94 °C (30 s.), annealing at 60°C (45 s.), and elongation at 72°C (45 s.).

The amplified product was visualized on 2% agarose gel electrophoresis under the Biosystems Genetic Analyzers imaging system (Applied Biosystems and Hitachi, Ltd., US).

The presence of a single 148-bp band either for wild type or variant allele in homozygous condition indicates successful amplification. While the presence of both bands within the same size (148-bp) represent the heterozygous condition [14].

The studied adolescents’ data were analyzed to detect the relation between serum 25(OH)D and PD (primary outcome). The secondary outcome measures the odds of PD in Asian adolescents with VDR TaqI polymorphism.

### Sample size

The required sample size for this study was calculated based on the number of adolescents between 12–18 years-old in Aktobe-Kazakhstan (27,972), prevalence of dysmenorrhea among adolescents (8–83%) and G Power software for sample size calculation (version 3.1.9.7, Düsseldorf; Germany) with 0.05 probability, 0.95% power, 0.5 sample size and student-t test for statistical analysis.

A sample size including ≥ 210 adolescents in two groups (105 in the study group and 105 controls) was enough to produce a statistically acceptable figure.

### Statistical analysis

The SPSS (Statistical Package for Social Sciences) version 25 (Chicago, IL, USA) was used for analysis of the collected adolescents’ data. The mean and standard deviation (± SD) were used to present numerical values. The number (n) and percentage (%) were used to present categorical values.

The student-t test and the Chi-square (x^2^) were used for analysis of the adolescents’ quantitative and qualitative data, respectively. The correlation analysis was done using the Pearson’s correlation coefficient (r) to detect the relation between serum 25(OH)D and PD. The MedCalc 20.106 (MedCalc. Ltd, Belgium) was also used to calculate the odds ratio (OR) of PD in Asian adolescents with VDR TaqI polymorphism. *P* < 0.05 considered significant.

## Results

Two hundred and five (205) adolescents between 12–18 years-old complaining of PD [study group (dysmenorrhea group)] were compared in this prospective study to matched controls (210 adolescents without dysmenorrhea) to detect the relation between serum 25(OH)D and PD, and the odds of PD in Asian adolescents with VDR TaqI polymorphism.

The studied-dysmenorrhea group and controls were matched with no significant difference regarding the mean age (14.8 ± 1.7 versus 15.2 ± 1.6 years, respectively), (*P* = 0.19), height (159.9 ± 3.28 versus 158.4 ± 3.7 cm, respectively), (*P* = 0.9) and weight (61.8 ± 6.9 versus 60.2 ± 6.4 kg, respectively), (*P* = 0.1). The studied-dysmenorrhea cases had significantly higher BMI compared to controls (24.2 ± 2.06 versus 23.9 ± 1.65 kg/m^2^, respectively), (*P* = 0.0007) Table 1.

**Table 1:** Characteristics of the studied-dysmenorrhea group compared to controls

Variable	Dysmenorrhea group (Number = 205)	Controls (Number = 210)	*P value* (95% CI)
Age (Years)	14.8 ± 1.7	15.2 ± 1.6	0.19 (-0.72, -0.4, -0.08)
Height (Cm)	159.6 ± 3.28	158.4 ± 3.7	0.9 (0.53, 1.2, 1.9)
Weight (Kg)	61.8 ± 6.9	60.2 ± 6.4	0.1 (0.32, 1.6, 2.9)
BMI (Kg/m^2^)	24.2 ± 2.06	23.9 ± 1.65	**0.0007**^**a**^ (-0.06, 0.3, 0.7)
25(OH)D (ng/ml)	16.17 ± 7.36	17.65 ± 6.36	**0.01**^**a**^ (-2.8, -1.5, -0.15)

Data presented as mean ± Standard deviation (SD) and Student t test used for statistical analysis

*BMI* Body mass index, *CI* Confidence interval

^a^Significant difference

### The relation between serum 25(OH)D and PD

**Fig. 1:**
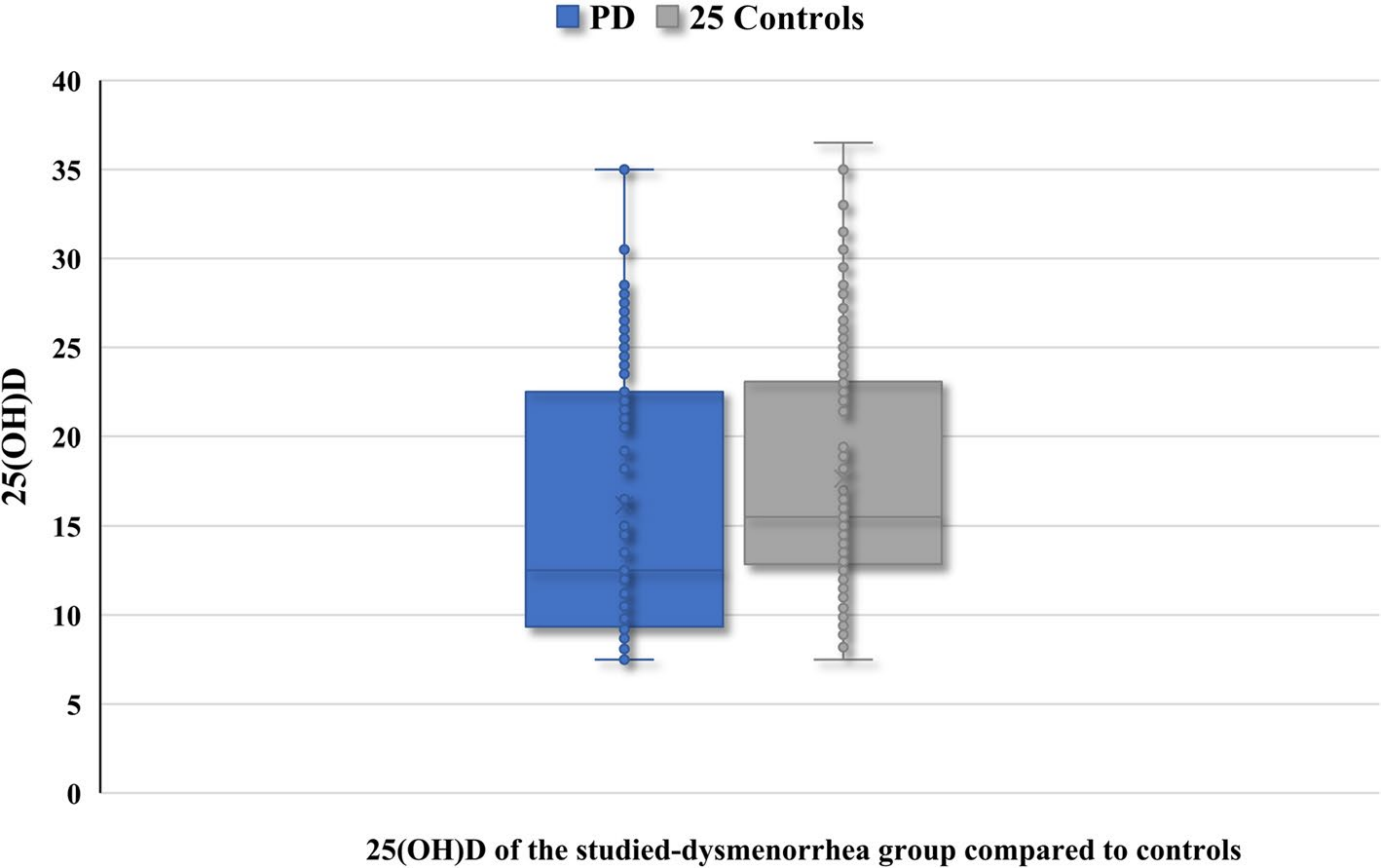
25(OH)D of the studied-dysmenorrhea group compared to controls. PD: Primary dysmenorrhea

The serum 25(OH)D was low in the two-studied groups, and it was significantly lower in the studied-dysmenorrhea group compared to controls (16.17 ± 7.36 versus 17.65 ± 6.36 ng/ml, respectively), (*P* = 0.01) Table 1 and Fig. 1.

**Fig. 2:**
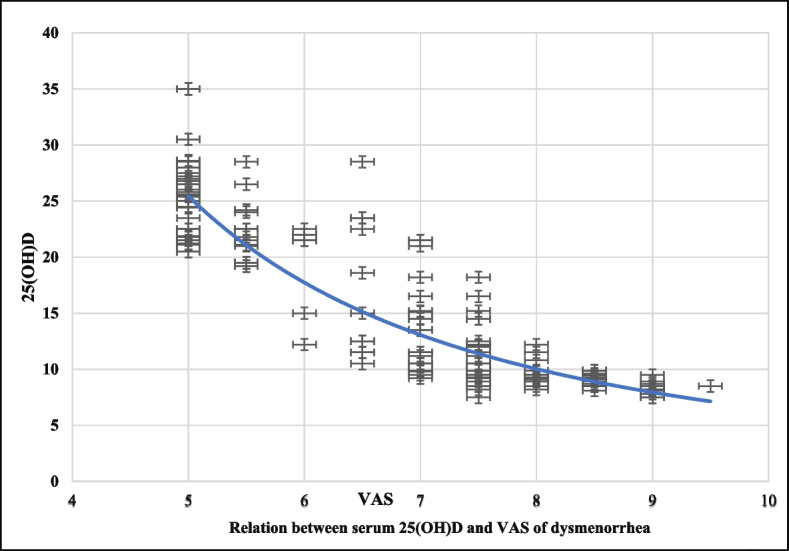
Relation between serum 25(OH)D and VAS of dysmenorrhea.VAS: Visual analogue scale

The correlation analysis showed a significant negative correlation between the serum 25(OH)D and VAS of dysmenorrhea (r = -0.9003, *P* < 0.0001) Fig. 2.

### The relation of VDR TaqI polymorphism to serum 25(OH)D and PD

The frequencies of the protective (T) and risky (t) alleles for the studied adolescents were 0.84 (348/425) and 0.16 (67/415), respectively according to the Hardy–Weinberg allele frequencies equation.

According to the protective (T) and risky (t) alleles, the VDR TaqI was divided into T/T (homozygous form), T/t (heterozygous form) and t/t (variant form) genotypes.

The frequencies of the T/T, T/t, and t/t VDR TaqI genotype for the studied adolescents were [0.214 (89/451), 0.624 (259/415) and 0.16 (67/415), respectively].

**Fig. 3:**
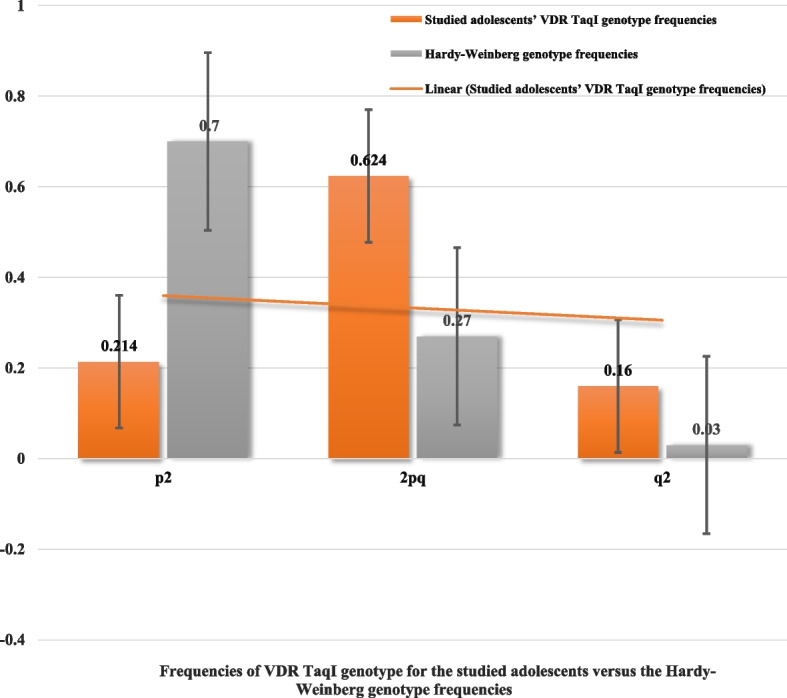
Frequencies of VDR TaqI genotype for the studied adolescents versus the Hardly-Weinberg genotype frequencies. VDR: Vitamin D receptor

The frequencies of the T/T, T/t, and t/t VDR TaqI genotype for the studied adolescents (0.214, 0.624 and 0.16, respectively) were statistically similar with no difference when compared to the Hardy–Weinberg genotype frequencies (0.7, 0.27 and 0.03, respectively), (*P* = 0, 0 and 0, respectively) Fig. 3.

The VDR T/t genotype was significantly more frequent in the studied-dysmenorrhea group [76.6% (157/205)] compared to controls [48.6% (102/210)], (*P* = 0.004).

The VDR T/T genotype was more frequent in the controls [39% (82/210)] compared to the studied-dysmenorrhea group [3.4% (7/205)], (insignificant difference, *P* = 0), and the VDR t/t genotype was more frequent in the studied-dysmenorrhea group [20% (41/205)] compared to controls [12.4% (26/210)], (insignificant difference, *P* = 0.07) Table 2.

**Table 2:** The VDR TaqI genotypes and serum 25(OH)D of the two-studied groups

Variable	Dysmenorrhea group (Number 205)	Controls (Number 210)	*P value* (95%CI)
**T/T**	7/205 (3.4%)	82/210 (39.0%)	*P* = 0
**25(OH)D** (ng/ml)	13.7 ± 1.6	14.7 ± 5.7	*P* = 1 (-2.7, -1, 0.7)
**T/t**	157/205 (76.6%)	102/210 (48.6%)	***P* = 0.004**^**a**^
**25(OH)D** (ng/ml)	16.7 ± 8.05	18.97 ± 6.7	***P* = 0.02**^**a**^ (-4.1, -2.3, -0.45)
**t/t**	41/205 (20%)	26/210 (12.4%)	*P* = 0.07
**25(OH)D** (ng/ml)	14.4 ± 4.1	21.4 ± 2.45	***P* = 0.004**^**a**^ (-8.61, -7, -5.4)

Chi-square (X^2^) test was used for statistical analysis when data presented as number and %

CI: Confidence interval. Data presented as number (n) and percentage (%) and mean ± Standard deviation (SD)

Student t test was used for statistical analysis when data presented as mean and SD. T/t: Heterozygous form

T/T: Homozygous form. t/t: Variant form. VDR: Vitamin D receptorSignificant difference

^a^Significant difference

**Fig. 4:**
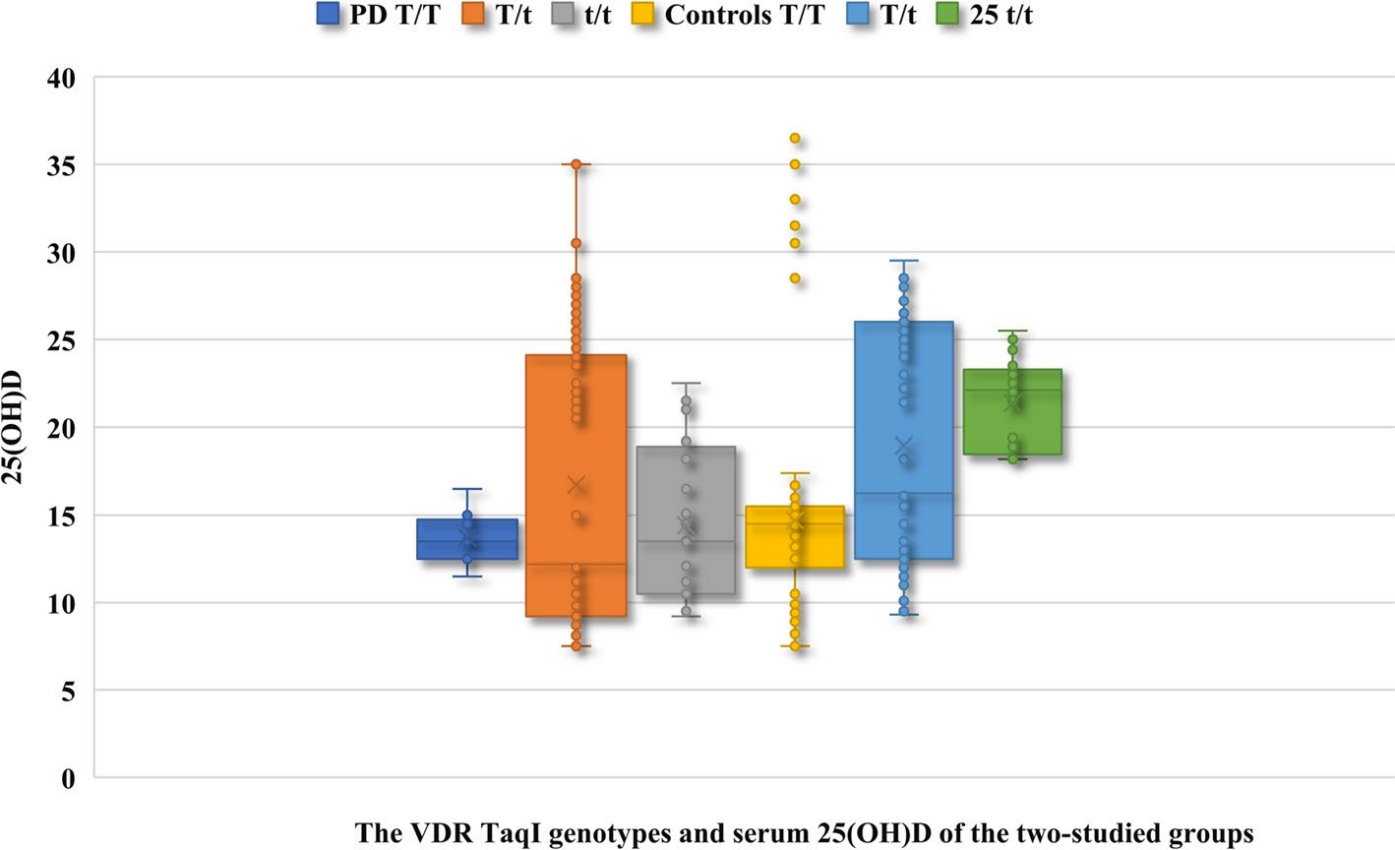
The VDR TaqI genotypes and serum 25(OH)D of the two-studied groups. PD: Primary dysmenorrhea. T/t: Heterozygous form. T/T: Homozygous form. t/t: Variant form. VDR: Vitamin D receptor

The studied-dysmenorrhea cases with VDR T/t and t/t genotypes had significantly lower serum 25(OH)D (16.7 ± 8.05 and 14.4 ± 4.1 ng/ml, respectively) compared to controls (18.97 ± 6.7 and 21.4 ± 2.45 ng/ml, respectively), (*P* = 0.02 and 0.004, respectively) Table 2 and Fig. 4.

The studied dysmenorrhea cases with VDR T/T genotype also had lower serum 25(OH)D (13.7 ± 1.6 ng/ml) compared to controls (14.7 ± 5.7 ng/ml), (insignificant difference, *P* = 1) Table 2 and Fig. 4.

The VDR T/t and t/t polymorphisms significantly increase the odds of PD (OR 1367.2, *P* < 0.0001 and OR 106.2, *P* = 0.001, respectively) Table 3.

**Table 3:** The odds of primary dysmenorrhea with different VDR TaqI genotypes

**Variable**	**Dysmenorrhea group** **(Number 205)**	**Controls** **(Number 210)**	**OR** [***P* value (95%CI)]**
**T/T**			
-Positive	7	0	15.9 [0.05 (0.9—280.4)]
-Negative	198	210	
**T/t**			
-Positive	157	0	1367.2 [**< 0.0001**^**a**^ (83.7—223,342.5)]
-Negative	48	210	
**t/t**			
- Positive	41	0	106.2 [**0.001**^**a**^ (6.48—1739.5)]
- Negative	164	210	

*CI* Confidence interval, *OR* Odds ratio, *T/t*, Heterozygous form, *T/T* Homozygous form, *t/t* Variant form, *VDR* Vitamin D receptor

^a^Significant difference

## Discussion

Increased PGDs is the most accepted aetiology of PD [6, 7]. The PGDs increase the amplitude and tone of uterine contractions [7]. The expression of VDR in the normal endometrium and ovaries [13], supports the role of Vit. D in local immunity and inflammatory cytokines regulation [14].

Vit. D metabolites [i.e., 25(OH)D and 1,25(OH)_2_D] reduce the inflammatory cytokines [i.e., interleukin-6 (IL-6) and TNF (tumor necrosis factor)] and uterine PGDs [17].

Therefore, two-hundred and five (205) adolescents between 12–18 years-old complaining of PD [study group (dysmenorrhea group)] were compared in this prospective study to matched controls (210 adolescents without dysmenorrhea) to detect the relation between serum 25(OH)D and PD, and the odds of PD in Asian adolescents with VDR TaqI polymorphism.

### The relation between serum 25(OH)D and PD

The serum 25(OH)D was significantly lower in the studied-dysmenorrhea group compared to controls (*P* = 0.01). The correlation analysis showed a significant negative correlation between the serum 25(OH)D and VAS of dysmenorrhea (r = -0.9003, *P* < 0.0001).

An observational study found a significant negative correlation between 25(OH)D and dysmenorrhea [16]. The same observational study found the Vit. D intake decreases dysmenorrhea and its associated symptoms [16].

A randomized controlled study reported Vit. D deficiency in dysmenorrhea with a significant negative correlation between the VAS of dysmenorrhea and serum Vit. D [25].

*Kucukceran *et al., [17] found the VAS of PD and the consumed NSAIDs significantly reduced after a single oral dose of cholecalciferol (300,000 IU) compared to placebo.

*Rahnemaei *et al., [6] randomized controlled trial reported significant reduction of menstrual pain and consumed analgesics after Vit. D supplementation.

Moreover, *Bahrami *et al., [26] found the high dose (50,000 IU cholecalciferol/week for 9 weeks) of Vit. D significantly reduces the dysmenorrhea and the premenstrual syndrome.

A systematic review reported a negative association relation between the serum Vit. D/calcium and severity of PD [12].

A recent randomized-controlled trial found the Vit. D supplementation could decrease the severity of PD and the consumed analgesics [27].

### The relation of VDR TaqI polymorphism to serum 25(OH)D and PD

The studied-dysmenorrhea cases with VDR T/t and t/t TaqI genotypes had significantly lower serum 25(OH)D compared to controls (*P* = 0.02 and 0.004, respectively).

The VDR gene is a protein-coding gene, located on 12q13.11 and expresses a receptor rs731236 that influences the Vit. D binding ability. Any mutation in the VDR gene may result in defective Vit. D binding and subsequent Vit. D deficiency and/or defective action [14].

The VDR polymorphism and the subsequent Vit. D deficiency [28, 29] or defective action [30, 31] was reported with different female reproductive disorders [32–34].

*Pekkinen *et al., studied the Vit. D binding protein (DBP) rs4588 gene and reported Vit. D deficiency in adolescents with DBP gene GC polymorphism [28].

*Amanzholkyzy *et al., studied the VDR polymorphism and found the VDR TaqI t/T polymorphism was associated with significantly lower serum 25(OH)D in Kazakh adolescents [29].

Additionally, a randomized double-blind trial found participants with VDR Taql TT/Tt polymorphisms were more responsive to Vit. D supplementation compared to participants with VDR Taql tt polymorphism [30].

Moreover, *Arabi *et al., [31] randomized trial found the VDR Taql polymorphism affects the adolescents’ response to Vit. D supplementation.

A literature review (containing retrieved data from 50 scientific sources) reported an association between Vit. D deficiency and different pubertal menstrual dysfunctions [32].

*Güleç Yılmaz *et al., [33] found the VDR *fok1* polymorphism increases the risk for dysmenorrhea, uterine fibroids, and heavy menses. *Mun *et al., [34] reported an increased risk of ovarian and breast cancers in women with VDR *fok1* polymorphism.

Although, the previous studies reported an association between VDR polymorphism and Vit. D deficiency and/or defective action [28–31]. The current study was the first study conducted in West Kazakhstan to detect the relation between serum 25(OH)D and PD, and the odds of PD in Asian adolescents with VDR TaqI polymorphism.

This study found the serum 25(OH)D was significantly lower in the studied-dysmenorrhea group compared to controls. The correlation analysis showed a significant negative correlation between the serum 25(OH)D and VAS of dysmenorrhea. The studied-dysmenorrhea cases with VDR T/t and t/t TaqI genotypes had significantly lower serum 25(OH)D compared to controls. The VDR T/t and t/t polymorphisms significantly increase the odds of PD.

### The relation between PD and BMI

Although, the BMI of the studied-dysmenorrhea cases was significantly higher compared to controls (*P* = 0.0007). The relation between BMI and PD is still inconsistent and controversial [35] and needs further research.

*Ju *et al., [36] reported higher risks of dysmenorrhea in underweight and obese participants. *Jiang *et al., [37] reported higher odds of dysmenorrhea in women with lower or higher BMI and *Gurdip *et al., [38] reported a significant relation between dysmenorrhea and underweight or overweight women. The controversial relation between BMI and PD, explains why adolescents with > 30 kg/m^2^ BMI were excluded from this study.

The limited number of studies investigating the relation between VDR TaqI polymorphism and PD was one of the limitations of this study. The relation between VDR TaqI polymorphism and adolescents’ PD needs to be confirmed in further studies. Adolescents refused to participate was another limitation of this study.

## Conclusion

The serum 25(OH)D was significantly lower in the studied-dysmenorrhea group compared to controls. The studied-dysmenorrhea cases with VDR T/t and t/t TaqI genotypes had significantly lower serum 25(OH)D compared to controls. The VDR T/t and t/t polymorphisms significantly increase the odds of PD.

## Data Availability

All data generated or analysed during this study were included and submitted for review with the study.

## Abbreviations

BMI: Body mass index
x^2^: Chi-square
OR: Odds ratio
IL-6: Interleukin-6
NSAIDs: Non-steroidal anti-inflammatory drugs
PCR: Polymerase chain reaction
PD: Primary dysmenorrhea
PGDs: Prostaglandins
SD: Standard deviation
TNF: Tumor necrosis factor
VAS: Visual analogue scale
VDR: Vitamin D receptor
Vit. D: Vitamin D
WHO: World Health Organization
WKMU: West Kazakhstan Medical University
